# Current Landscape and Open Questions on Adjuvant Therapies in Melanoma

**DOI:** 10.5826/dpc.11S1a165S

**Published:** 2021-07-01

**Authors:** Vincenzo De Falco, Stefania Napolitano, Luigi Pio Guerrera, Teresa Troiani

**Affiliations:** Medical Oncology, Department of Precision Medicine, Università degli Studi della Campania “Luigi Vanvitelli”, Napoli, Italy

**Keywords:** locoregional melanoma, stage III melanoma, adjuvant therapy

## Abstract

Melanoma is a form of skin cancer that is frequently diagnosed at early stages. In most cases, surgical resection is curative. In case of thicker melanomas (> pT1b) without clinical or instrumental evidence of metastasis, a sentinel lymph node biopsy is recommended for staging purposes. If the lymph nodes are the only site of disease (macroscopic or microscopic> 1mm), configuring stage III, the international guidelines recommend the use of adjuvant therapy with checkpoint inhibitors (nivolumab or pembrolizumab) or targeted therapies (dabrafenib plus trametinib). These drugs have shown a significant increase in recurrence-free survival, although some doubts and open questions remain. Specifically, none of the available treatments has shown a clear benefit in the overall survival rates, the advantages they give in stage IIIA are not well known, and finally there are still no prospective clinical studies identifying the best approach to continue the therapeutic process in case of relapse. Furthermore, there are new opportunities opening up with the upcoming results of the neoadjuvant trials that could revolutionize the treatment of clinically evident stage III melanoma.

## Introduction

Melanoma is the deadliest of skin cancers and occurs primarily the elderly, still, it is one of the most common cancers diagnosed in young adults, particularly in women [1]. About 50% of patients with cutaneous melanoma harbor a mutation in exon 15 (codon 600) of *BRAF* proto-oncogene, conferring a worse prognosis [2]. According to the new 8th edition of the American Joint Committee on Cancer (AJCC) staging, patients with early-stage (I–II) have an overall favorable prognosis, whereas patients with stage III melanoma have a rather heterogeneous prognosis [3]. The discovery of immune checkpoint inhibitors and targeted therapies (TT) revolutionized the treatment scenario of metastatic melanoma and with the latest evidence these drugs were also added in adjuvant setting. In this work, we will review the state of art and the unresolved questions of adjuvant therapy. Finally, we will examine future directions for stage III cutaneous melanoma.

### Immunotherapy (IT)

Until few years ago, only interferon-α (IFN-α) showed a survival benefit in this setting, although it had a very modest efficacy and was limited to ulcerated melanoma [4]. Anti-programmed cell death-1 (anti-PD-1) antibodies such as nivolumab and pembrolizumab and anti- cytotoxic T lymphocyte-associated antigen 4 (anti-CTLA-4) antibodies such as ipilimumab clearly exhibited a benefit in terms of progression free survival (PFS) and overall survival (OS) in patients with metastatic melanoma [5]. For this reason, several trials have evaluated the efficacy of these drugs in reducing the risk of relapse in stage III radically resected melanomas. In 2015, EORTC 18071 trial evaluated ipilimumab at a dose of 10mg/kg versus placebo for up to 3 years in patients who had undergone complete resection of stage III melanoma [6]. Both recurrence-free survival (RFS) and OS were significantly superior in the ipilimumab group compared to placebo group at the cost of very high percentage of serious adverse events (5 patients died for immune-related toxicities). Ipilimumab was therefore considered too toxic and was not approved by the European Medicines Agency (EMA) in patient populations who are potentially cured with surgery alone. On the other hand, CheckMate-238 trial tested nivolumab 3mg/kg vs ipilimumab 10mg/kg for up to 1 year in stage IIIB-C (81.3% of study population) and stage IV radically resected melanomas [7]. The 4-year RFS was 52.4% for the nivolumab group vs 24.1% for the ipilimumab group, with a Hazard Ratio (HR) of 0.71 (P=.0003) [8]. Most frequent adverse events were fatigue, diarrhea, pruritus, and rash but only in 14.4% of cases, these were grade 3–5. Nonetheless, the KEYNOTE-054 trial compared pembrolizumab 200mg versus placebo in stage IIIA-B-C radically resected melanoma: 3.5-year RFS was 59.8% versus 41.4%, respectively (HR 0.59, p<.001). Adverse events were similar to those reported with other anti-PD1 inhibitors [9]. Very recently, the last update of the S1404 trial was presented, in which pembrolizumab was compared with 1 year of high dose interferon or up to 3 years of ipilimumab in radically resected stage III or IV melanoma: HR for RFS was 0.74 (p<0.001) [10]. After these strong evidences, nivolumab and pembrolizumab were approved by EMA as adjuvant therapy for all stage III melanomas (nivolumab also for radically resected stage IV).

Finally, also the combination of nivolumab and ipilimumab has been tested in adjuvant settings in IMMUNED trial and CheckMate-915 trial with conflicting results. In the first case, a German phase II trial, patients with radically resected stage IV melanoma were randomly assigned to nivolumab+ipilimumab or nivolumab alone, or placebo. HR for recurrence for the doublet group vs placebo was 0.23 and median RFS was not reached after median follow-up of 28.4 months [11]. On the contrary, phase III CheckMate-915 examined adjuvant nivolumab vs combination of nivolumab and ipilimumab in resected stage IIIB-D or IV melanomas, but it did not meet its endpoint [12]. These results might be due to the different study population and to the different dose/frequency of ipilimumab, nonetheless further studies are needed.

### Targeted therapy (TT)

As immunotherapies, BRAF and MEK inhibitors represented a turning point for the treatment of BRAF mutant metastatic melanomas with very high response rates and a significant benefit in terms of PFS and OS. Regarding the efficacy in the adjuvant setting, COMBI-AD trial tried to show the efficacy of these drugs also in the adjuvant setting. It compared dabrafenib (BRAF inhibitor) 150 mg twice daily plus trametinib (MEK inhibitor) at a dose of 2 mg once daily, versus placebo in stage IIIA-B-C melanoma. In the last update, 3-years RFS was 58% in the experimental arm and 39% in the placebo arm (HR 0.47 p<0.001) [13]. Adverse events were represented by pyrexia, fatigue, nausea, headache, chills, diarrhea, arthralgia, and rash. These were of grade 3 to 5 in 41% of cases. However, also dabrafenib plus trametinib became a valid option as adjuvant therapy.

### Open Questions on Adjuvant Therapy and How to Manage Recurrences

Despite the undoubted effectiveness of these therapies, a number of open questions still remain open. These concern for instance the timing of their use and the risk/benefit ratio in some subgroups of patients. First of all, there is still no evidence regarding the benefit in survival rates: although ipilimumab had already demonstrated an OS advantage vs placebo, in the CheckMate-238, following a 48 months follow-up there are no differences in OS between nivolumab and ipilimumab (78% vs 77%, HR 0.87 p=0.315)[8]. However, fewer events than expected occurred in the trials, so it is underpowered. Also, for pembrolizumab in S1404 no benefit in OS was observed [10]. Moreover, in the COMBI-AD trial the statistical significance did not reach the prespecified target of p=0.000019 (3-years OS: 86% vs 77%, HR 0.57 p=0.0006) [13]. Definitive data of these 2 studies and of KEYNOTE-054, the only study in which a cross-over between treatments was allowed, will clarify if starting the therapy at the time of relapse affects survival rates.

A second important aspect is that all these studies started before the definitive data of Multicentre Selective Lympadenectomy Trial II [14] and the German Dermatologic Cooperative Oncology Group-selective lymphadenectomy trial [15], that did not report an improvement in melanoma specific survival (MSS) for complete lymph node dissection versus periodic ultrasonographic surveillance in patients with positive sentinel lymph node. This suggests that the study population does not correspond to patients treated in current clinical practice.

Moreover, the new edition of AJCC staging was approved and the main changes concerned stage III: stage IIID was added, and the subgroups were re-distributed. More in detail, stage IIIA now includes T1a-b N1-2a and T2a N1-2a [16]. In the adjuvant trials, only patients categorized as stage IIIA with nodal metastases >1mm (CheckMate-238 did not include them), were included. Furthermore, patients enrolled in these trials with positive SLN have had lymphadenectomy, indicating that some of the stage IIIA may be up-staged. On the other hand, in clinical practice, several patients without nodal dissection could be downgraded to IIIA (for example if they have metastatic non-sentinel lymph nodes). However, taking into account the high melanoma specific survival in this stage (80%-93%) [17], and the risks of durable and serious adverse events, adjuvant therapy should be carefully discussed with these patients [18].

Finally, an unmet need that originated from adjuvant trials is the management of relapses during and after treatment. There is in fact a lack of prospective randomized studies investigating this question, as only retrospective experiences are reported. What we know is that the majority of recurrences are with distant metastases (including locoregional+ distant metastases) and they are mostly on-treatment during anti-PD-1 therapy [19] and after treatment with targeted therapies [20, 21]. This observation led to support the idea that treatment with BRAF- and MEK-inhibitors should be prolonged to more than a year (2–3 years?) to improve its efficacy. However, when the relapse occurs during adjuvant therapy (or within few months from its conclusion), it is good practice to switch to another treatment, particularly in BRAF mutant patients (IT→TT and TT→IT). On the contrary, a rechallenge approach, adopting the same drugs, when the relapse occurs off treatment, could be a good option because of good response rates, especially for TT. Nevertheless, data from pembrolizumab rechallenge in the KEYNOTE-054 study, performed on patients who recurred after 6 months from the completion of adjuvant therapy, were very disappointing [22]. Furthermore, radical surgery followed or not by systemic adjuvant therapy, should be done when recurrence is locoregional and when radical surgery is achievable.

### A Step Forward

There are several ongoing trials trying to solve the open questions for the management of locoregional melanoma. One of these issues concerns adjuvant therapy for melanomas without involvement of lymph nodes: paradoxically, 5-year survival of stage IIB (87%) and IIC (82%) is worse than stage IIIA (93%). KEYNOTE-716 and CheckMate-76K will compare the efficacy of pembrolizumab and nivolumab, respectively, versus placebo in these patients. Results are expected for 2023–24.

A closer change in clinical practice will probably come from neoadjuvant studies for clinical stage III melanoma. Up to now, the relapse rate for radically resected melanoma with nodal macro metastases was 40% at 2 years with immunotherapies and 40% at 3 years with targeted therapies (without considering 15–20% of patients in the trials recurred during the screening period before the start of adjuvant therapy) [23]. Neoadjuvant therapy could improve outcome from surgery, could personalize adjuvant treatment based on treatment response, and could safely provide tissue for analysis of resistance mechanisms from those who do not have a pathological response. For this reason, in the last years several trials have evaluated this strategy and a recent pooled analysis summarized the results of 6 of them (2 with targeted therapy, 4 with immunotherapy) [19]. In particular, pathological complete response (pCR) was found to be a good surrogate of RFS and OS. pCR rate was 39.7% in the whole cohort: worst results were found for single agent nivolumab (pCR 20%), while similar outcomes were found for dabrafenib+trametinib (47%) and for nivolumab+ipilimumab (42.7%). The RFS was similar between combination immunotherapies and targeted therapies after 1 year (84% vs 75%) while a significant difference was seen at 2 years (80% vs 47%). Furthermore, with nivolumab+ipilimumab, impressive OS were achieved in patients who obtained a pCR, or a near pCR, or a partial response (about 2/3 of patients) reaching 99% after 2 years. Despite this very promising result, larger studies are needed to confirm these findings and to clarify other open questions such as understanding the mechanisms underlying the relapse in 21% of patients with pCR to targeted therapy.

## Conclusion

The efficacy of immunotherapy and targeted therapy as adjuvant treatment in stage III melanoma is unquestionable. Something could change soon when the overall survival results will be consolidated and when data on neoadjuvant therapy will be more consistent. To date, there is no evidence that one type of treatment among those approved is more effective than another. For this reason, personalized treatment must be based on the clinical-pathological characteristics of the disease, on patient compliance, and on comorbidities, taking into account the side effects of each drug.
